# Supplementary material for: Climate drivers of malaria at its southern fringe in the Americas
Source: PLoS One. 2019 Jul 10;14(7):e0219249. doi: 10.1371/journal.pone.0219249 (PMC6619762; doi:10.1371/journal.pone.0219249)
Supplement: S1 Appendix — (PDF) [file pone.0219249.s001.pdf]

Supporting Information for: Climate drivers of malaria at its southern fringe in the Americas

Karina Laneri, Brenno Cabella, Paulo Inácio Prado,
Renato Mendes Coutinho, Roberto André Kraenkel

S1 Appendix

Relapse cases of *P. vivax* in Argentina have not been well documented. For other subtropical regions with low transmission, the period between relapses can be very long, from 8 months to years [1, 2, 3]. Also in low transmission settings the number of relapses due to a primary infection tend to be small, from 1 to 4 relapses [3]. In Argentina there are no data on relapse times—patients that are treated after the first infection are not followed up to record when they experience relapses. Parasitemia levels have not been measured either. Even if *P. vivax* parasitemia levels were much lower than in the case of *P. falciparum*, parasitemia increases with relapses but immunity also increases.

As a preliminary exploration, we estimated the timing of relapse cases by assessing the autocorrelation of the number of malaria cases time series [2]. Even though there are few cases in Argentina and a high associated measurement error, we obtained a slight autocorrelation increase from 4 to 10 months, which is in agreement with previously reported results of *P. vivax* in India [2]. For a more accurate measure of relapse times, future studies would be needed to follow up cases.

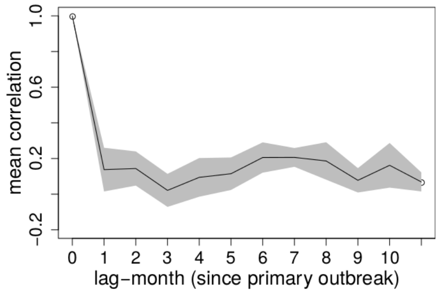

Fig S1. Malaria cases auto-correlation. Averaged auto-correlation of *P. vivax* malaria cases (solid line) and associated error (gray) for each of the transmission months (November-April) with malaria cases in the subsequent 12 months. It slightly increases between 4 to 10 months from the primary outbreak, associated with relapses period. The average was done over all years.

References

[1] Organization WH. World malaria report 2017. Geneva. Licence:CC BY-NC-SA 3.0 IGO.; 2017.

[2] Roy M, Bouma MJ, Ionides EL, Dhiman RC, Pascual M. The Potential Elimination of Plasmodium vivax Malaria by Relapse Treatment: Insights from a Transmission Model and Surveillance Data from NW India. PLOS Neglected Tropical Diseases. 2013;7(1):1–10. doi:10.1371/journal.pntd.0001979.

[3] Kondrashin AV, Morozova LF, Stepanova EV, Turbabina NA, Maksimova MS, Morozov EN. On the epidemiology of Plasmodium vivax malaria: past and present with special reference to the former USSR. Malaria Journal. 2018;17(1):346. doi:10.1186/s12936-018-2495-y.
